# Book Reviews

**Published:** 1822-02

**Authors:** 


### CRITICAL ANALYSIS
OF
### ENGLISH AND FOREIGN LITERATURE
RELATIVE TO THE VARIOUS BRANCHES OF
### Medical Science.

Quæ laudanda forent, et quæ culpanda, vicissim

Illa, prius, cretâ; mox hæc, carbone, *notamus*.—PERSIUS.

### DIVISION I.

### ENGLISH.

ART. I.—*A Treatise on Diseases of the Nervous System. Part the First: comprising Convulsive and Maniacal Affections.* By J. C. PRICHARD, M.D. late of Trinity College, Oxford; Fellow of the Linnean and Wernerian Societies, &c.; Physician to St. Peter's Hospital and the Bristol Infirmary. 8vo. pp. xii. 425. T. and G. Underwood. London, 1822.

SINGULAR as this book will, indubitably, appear to many of our readers, it contains, nevertheless, much original and valuable information. There are, it is true, sundry odd ideas in it; but we meet also with many very excellent observations, in its various subdivisions, which are either new or better told than by any old or recent writer.

The principal objects of Dr. Prichard's work seem to be those of rendering the history of diseases of the nervous system somewhat more accurate and precise than it has hitherto been; and of improving the treatment of those complaints.

Dr. Prichard has acquired, in the course of a pretty extensive practice, as physician to an hospital, the conviction that disorders of the nervous system are, in the majority of cases, secondary and sympathetic affections; that they are often, at least, symptoms of some latent disease in another part of the constitution, and, particularly, in the state, either temporary or permanent, of those organs which are subservient to the natural functions.

On the adoption of these principles depends Dr. Prichard's new mode of discriminating nervous affections into classes, corresponding to the nature of the primary diseases of which they are symptoms. Nothing, in our opinion, can be clearer, or more systematically correct, than this mode of reasoning *à priori*, and upon *data* founded, chiefly, on observed phenomena regularly recorded.

What the author states respecting the treatment of the diseases in question, seems, also, to follow, naturally, out of his peculiar manner of viewing the subject.

"A similar method likewise holds out the best expectation of improvement in practical treatment; for it is obvious that the cure of such diseases, in so far as they are sympathetic, must be founded on, and bear a relation to, the nature of the primary affection. Some of the diseases, of which I purpose to treat, are commonly regarded, at least in cases of long duration, as almost incurable by any efforts of human art. So repeatedly have medical practitioners been disappointed in the trial of various remedies proposed for the cure of these disorders, that any new suggestion of this kind is justly regarded with suspicion; yet numerous instances occur in which nature, in some period or another of life, effects a cure. The diseases are found to cease in consequence of some spontaneous change which takes place in the state of the constitution. If medical practitioners, instead of hunting after specific remedies, carefully directed their attention to trace the method by which these natural terminations are brought about, or to ascertain the process of those constitutional alterations, in consequence of which the diseases alluded to disappear, it is probable that they might be enabled, in some instances, to imitate the salutary operations of nature."

Where the author himself acknowledges that his work is confused and unsystematic in its arrangement, we cannot be expected to give either a very concise or a clear account of its contents. We shall endeavour, however, to catch as much of the unity of Dr. Prichard's thoughts, as will enable our readers to judge both of the nature and value of his production.

The volume, as the title of it, indeed, announces, is dedicated to the consideration of convulsive and maniacal affections; but it likewise embraces some physiological and metaphysical observations on the functions of the nervous system generally; with a pathological survey of the diseases incidental to that system,—such as apoplexy, paralysis, epilepsy, mania, vertigo, tremor, somnambulism, chorea, and hysteria. Of all these complaints, however, epilepsy and mania are the only two on which the author has dilated at full length, and respecting which he has brought forward a great mass of facts and interesting observations, supported or illustrated by cases, in which the peculiar views, as well as the peculiar treatment, of the author are made manifest.

The consideration of epilepsy and mania, in fact, form the body of the work, and to them we will direct our principal attention.

We must not, at the same time, omit to say a few words on Dr. Prichard's physiological views of the functions of the nervous system, as we find them developed in the first chapter.

ANIMAL AND PHYSICAL FUNCTIONS.—The author has included under the denomination "physical" those functions inherent in the living body, which physiologists have generally distinguished by the two appellatives of *natural* and *vital*, and which are subservient to growth and merely corporeal life; while he considers the *animal functions* to be those which are dependent on the existence of a nervous system.

The former, or the "physical functions," are common to all orders of organized beings; the latter are peculiar to animals. In the physical functions, we can observe a connexion between the result or end obtained, and the means provided for its accomplishment. In the animal functions, on the contrary, we are far from perceiving the connexion between the instrumental operation and the effect intended or produced. What relation, for example, or common property, is there between the movement of a fibre and a sensation or thought?

In the second section of the first chapter, Dr. Prichard enters into an examination of the intellectual faculties, in respect to their connexion with the operation of the nervous system; and, although we apprehend that the subjects of sensation and perception, memory, association of ideas, imagination, and ratiocinating faculty, have been treated in a superior and more masterly style by Dugald Stewart, we shall not be found backwards in admitting a fair portion of merit in Dr. Prichard for this part of his investigation. As illustrative of that singular, yet inexplicable, faculty of the mind, memory, Dr. Prichard relates the following anecdote, communicated to him by Dr. Stock, of Clifton, who had heard it related by Dr. Rush, of Philadelphia, as a fact of which he had personal knowledge.

"A student at an university in the United States, who, as I may observe, is now one of the most respectable clergymen in that country, possessed a tolerable share of classical knowledge, when the consequences of a fever, which affected his brain, deprived him entirely of his former acquisitions. In fact, he had now become so ignorant that he was not only unable to read a Latin book, but even knew nothing of the grammar. When he had regained his bodily health, being of a persevering disposition, he began again the first rudiments: every thing was quite new to him; he passed through the accidence and syntax in his grammar, and was learning to construe, when, one day, as he was making a strong effort to recollect a part of his daily lesson, the whole assemblage of ideas, which he had formerly acquired and lost, suddenly re-appeared to his mind, and he found himself able to read and understand the Latin authors, as he had done before his illness."

The same inquiry is pursued by the author, with regard to other classes of mental phenomena, in the third section; including pathemata, embracing the consideration of passions, active principles, propensities, affections, and volition, with remarks on the relation they may be supposed to bear to the nervous system.

In a fourth section, Dr. Prichard shortly recapitulates all he has said on the physiology of the nervous system, and debates the following question: Is the nervous system the organ of any of the physical functions? That he is inclined for the negative will appear evident from the perusal of a short extract.

"This question seems to me to be settled, beyond all controversy, by an argument derived from some imperfect specimens of human organization; I mean children which have been born without brains, which, nevertheless, were possessed of the function of secretion, and endowed with irritable muscles. There are even well-authenticated relations, by which we learn that a human fetus can be formed, possessed of both these properties, and yet destitute of all rudiments of the nervous system."

On this subject, however, Dr. Alison's very able paper, in which he has placed, in a clear point of view, the arguments tending to prove that secretion and irritability are independent of the nervous system, must take the lead of our author's observations upon the same subject.

There are two notes, A and B, to the first chapter, which, if they be not instructive, are certainly curious.

The diseases incident to the nervous system are pathologically investigated in chapter the second. The resources we possess for conducting this investigation, may be mentioned under three heads, the most important being morbid anatomy. It would appear that many of the diseases of the class *neuroses* are more nearly connected, with respect to their causes and the morbid conditions in which they consist, than most of the disorders that are classed together in other departments of nosology. Thus, for instance, the affinity between apoplexy and paralysis is so apparent, that we continually see them passing into each other, as if dependent upon different degrees of the same morbid condition. The connexion of apoplexy with epilepsy has been noticed long ago, and many facts have been recorded by Morgagni, tending to illustrate the relations of these disorders. Epilepsy, though a distinct disease from apoplexy and palsy, is, in its relations, very near to both. Persons, who have partially recovered from a recent apoplexy, are often assailed by convulsions, which display most of the phenomena of epilepsy. On the other hand, persons, who fall victims to repeated fits of epilepsy, perish under all the symptoms of apoplexy.

With mania, apoplexy and hemiplegia display a like affinity. Maniacs are very subject to expire suddenly under an attack of apoplexy. Insanity, however, is still more intimately connected with epilepsy. Vertigo is sometimes a distinct disease, but it very frequently happens that vertigo is the harbinger of some more grievous malady.

Fits of tremor, and attacks of partial convulsions, bear some relation to epilepsy; and the connexion in pathology between chorea and paralysis, will be denied by few who have seen much of either of those complaints. Indeed, conversions of chorea, epilepsy, and paralysis, are by no means rare occurrences; and the author, on this point, relates the case of a child, in which there had been the following remarkable succession of symptoms.

"Three years ago the child laboured under whooping-cough, and the fits of coughing threw her into paroxysms of tetanic epilepsy. These symptoms subsided after continuing some months. She was next seized suddenly with a slight hemiplegia of the right side, from which she nearly recovered, but is now troubled with chorea, which agitates, though in no very severe degree, the limbs of the same side."

Hysteria, Dr. Prichard observes, is a disease which, in time, puts on the form of almost every individual distemper of the class under consideration. According to Sydenham's remarks, it is perfectly proteiform.

"The mutual conversions of disorders of the nervous system leads us to one conclusion, which is important with respect to the pathology of these complaints. Diseases which are so nearly allied as to be liable frequently to supersede or pass into each other, and at other times to co-exist in the same individual, must, as it should seem, when they have their seat in the same structure, depend on similar deviations from the healthy condition of the system. The predisposing circumstances, or remote causes, are indeed various, even in the instance of one and the same disorder; but the particular condition of the organic structure, from which the phenomena of the disease immediately result, is probably always similar in the same complaint; and in cases which, though they do not come under the same nosological definition, are yet connected by their frequent transitions and combinations, it may be presumed that the causes differ from each other only in slight modifications."

The connexion of nervous disorders with each other, is not the only point which Dr. Prichard endeavours to establish. In section fourth of the second chapter, he clearly defines the affinity existing between disorders of the nervous fabric and several diseases of the physical functions; and, although it be acknowledged that the pathology of diseases, whether of the nerves simply, or of the nerves and certain physical functions conjointly, be at present imperfect, the author is persuaded that, among those cases of nervous or cerebral diseases which are consequent upon irregularities in the natural or vital functions, a great majority will be found to depend upon actual, and often organic, disease in the brain or some other part of the nervous system.

Having established the fact, (though not a new one, truly,) that a relation exists between nervous diseases and complaints of the natural functions, Dr. Prichard proceeds to illustrate the doctrine by a considerable selection of cases and observations, which are distributed agreeably to the following arrangement:

"In one department, those cases of nervous diseases will be placed which originate in a suppression, tardy appearance, or other deficiency, of the periodical functions of the uterine system. A second class will include those instances of similar disease which are the result of torpor, or irregular action, in the intestinal canal, or of disorder in the functions of the stomach. Another division will consist of some cases in which morbid affections of the brain and nervous system are connected with disease of the liver. A fourth class may comprehend disorders of the animal functions, which may be termed idiopathic, since they arise in consequence of the operation of causes which act immediately on the functions of the nervous system, or induce primary disease in the structure of the brain. To this class belong, for example, those cases of madness and of epilepsy which arise from the influence of mental emotions,—as grief, terror, and the like. Another class of nervous disorders appear to depend on diseases of the heart, whether of function or of structure. Several cases of this description have casually fallen under my own observation, but I am afraid they are not sufficiently numerous to establish the fact of this connexion in a manner satisfactory to all my readers. Another division of diseases, distinct from all the foregoing, are those which arise from the metastasis of inflammatory disorders,—such as rheumatic and cutaneous inflammation, and the inflammation of serous membranes."

A note (C,) on the doctrine of particular determinations of blood, follows the conclusion of this chapter.

We now come to the more practical part of Dr. Prichard's work, which we have perused with great satisfaction.

A general description of epilepsy is continued in the third chapter, which is subdivided into—Section 1, giving the definition and nosological distinction of epilepsy; section 2, embracing an outline of the history of that disease,—the variations occurring in its symptoms,—its terminations and consequence. The pathology of epilepsy is discussed in the succeeding section; and, lastly, two cases from the author's own practice, and two others communicated by Dr. Laird, are brought forward in a note (D,) to illustrate the preceding observations.

Chapter fourth opens with an outline of the history of madness, of which it professes to give a general description, which may be summed up in the author's own words,—that "madness consists in mistaking the ideas of reverie for the impressions of memory and reflections." The same subject is continued in the second section; where the author, also, endeavours to show that every variety of madness can be reduced to the foregoing description.

A most important question arises out of the consideration of this interesting subject. What is the pathology of the brain in madness?—It has been a common complaint among morbid anatomists, that nothing has been found in the brains of maniacs sufficiently distinct to elucidate the disease. Dr. Prichard, who has briefly discussed this subject, thinks that all that can be expected to be found in maniacal complaints, are the common vestiges of increased vascular fullness, whether inflammatory or congestive; for, to the effect of increased vascular action, the author thinks all the phenomena, usually found in maniacs, must be referred.

The subjects treated next by Dr. Prichard, are of the first importance to a certain class of practitioners. The epileptic and maniacal cases depending on the state of the uterine functions, have hitherto attracted but little of the attention of the profession, and we were pleased to find our author had entered fully on their consideration. A short section, giving an outline of the history of uterine epilepsy, will be read with interest. We have had occasion to attend two or three important cases of this disease, occurring during gestation, and we may, perhaps, feel some partiality for the subject, from interested motives. Certain it is, that there are not more perplexing cases in the whole range of obstetrical medicine, than those of the nature to which we allude; nor any that are more likely to prove rebel to the best and most approved methods of treatment.

We shall pass over the fifteen cases related by the author, in many of which our readers will find much that is worthy of commendation. But by far the most important part of the present subdivision of Dr. Prichard's work, is that which relates to the treatment of the disease above mentioned. His observations on that subject are particularly deserving of notice; and we confess to have derived information from a perusal of the fourth section of the fifth chapter, which is novel to us, and on which we shall not fail to act on the earliest opportunity. We invite our readers to satisfy themselves of the truth of our remarks.

Uterine epilepsy may take place under different circumstances of the uterine functions. It may depend on a sudden disappearance of the catamenia; or on a laborious, scanty, and painful menstruation.

I. In epilepsy arising from amenorrhœa, the practical indications are—1, to relieve the morbid determination to the head; 2, to restore the natural determination to the uterine system; 3, if the latter object cannot be accomplished, to bring the constitution into a state in which the injurious effects of amenorrhœa are felt in a less degree. Abstraction of blood is one of the most important resources we possess for fulfilling the first indication. It has occasionally happened that menstruation has speedily followed the use of the lancet. The warm bath and friction will promote greatly the beneficial effect of bleeding. Stimulating glysters, blisters applied to the sacrum, and the emmenagogues, may be had recourse to, in their turn.

"If these measures succeed in producing a free and copious flow of the catamenia, the head becomes quickly relieved, the fits cease, and the health of the patient is, for the present, restored. But, if they fail of exciting the action of the uterine system, they generally relieve and often remove, the epileptic tendency, by diminishing plethora, and giving rise to some new determination."

Should the efforts made to restore menstruation prove abortive, all that remains to be attempted is to fulfil the third indications; for doing which Dr. Prichard has laid down many very judicious rules and instructions.

Of the five cases of epilepsy from amenorrhœa adduced by Dr. Prichard, we shall take leave to quote the third, as it is strongly illustrative of the effect of bleeding in that complaint.

"Sarah Boon,* æt. 20. Admitted in-patient, June 15, 1820. A girl of sanguine temperament, rather full habit, who has never been regular with respect to the catamenia; but has had, at distinct intervals, a slight appearance. About seven or eight months ago, she became subject to epileptic fits, which have continued to trouble her since that time.

"She has two or three fits every day, which sometimes continue an hour or more. She sometimes knows when they are coming on by a pain in her head, but more frequently they attack her without any premonitory symptom. Formerly she used to struggle while under the

* "This was a patient of Dr. Carrick's, the senior physician to the Infirmary; I had no part in the management of the case."

fit, now she lies quite still. Bowels generally regular.—Fiat ven. sec. et fluant ℥ xvj. Pulv. cath. o. n. Mist. aper. ter in die.

"16.—The blood is not inflamed. Pulse 94, and full. Bowels confined.—Repet. ven. sec. et fluant ℥ xvj. Mist. cath. 4ta. quâque horâ donec alvus fusa sit.

"17.—The blood not buffy. Fits as frequent as ever. She was now seen under one: lying quite still, breathing easily; with a pulse of 90; looking as if asleep: the pupils contracted on admission of light.—Repet. V. S. ad ℥ xvj. Epispast. inter scapulas.

"19.—She thinks herself rather better; but the fits are as frequent as ever.—V. S. ad ℥ xvj.

"20.—She was not bled yesterday, and had two fits, accompanied by some convulsions. She says she is better.—V. S. ad ℥xvj.

"21.—Only half the quantity of blood ordered was taken, because she had a fit at the time, and more could not be obtained. She says she is better.—V. S. ad ℥xvj.

"22.—Not yet bled. She had but one fit yesterday, which lasted about the usual time: this is the only day when she has had but one. She feels herself much better; has no head-ache. Her pulse is 104, full; her tongue clean.

"23.—She was bled yesterday, and had no fit all the day: she feels her head much relieved. Pulse 110, and full.

"25.—No fit yesterday. Some little pain in the head.

"26.—She has had no fit since the 21st. No appearance of the catamenia. Swelling of the right arm, resembling phlegmasia dolens. It has been poulticed, and is to-day better.

"28.—Her arm is still affected. She has had no fits.

"*July* 8.—No fits; no catamenia. Phlegmasia of the right leg; skin red. It is to be poulticed.

"12.—Redness of the leg gone: leg still swelled. She is to bandage it, and use a spirituous lotion. Takes pil. cath. o. n., and mist. cath. ter in die. No catamenia.

"25.—Leg swelled, and painful up to the knee. Pulse quick, and rather full.—V. S. ad ℥ xvj.

"28.—Felt better for the bleeding. Her legs now both swelled; swelling painful and hot, and pits a little. Bowels open. She feels well, except her legs.

"*Sept.* 7.—Her legs somewhat improved under the use of leeches and blisters, and purgative medicines. No fits; no catamenia.

"29.—Swelling almost gone. No fits; no catamenia.

"Made an out-patient. I saw her repeatedly many months afterwards, and found that she continued free from her fits, and in good health, except that the pains in her legs still troubled her occasionally.

"*Observations.*—The effect of bleeding and evacuations in uterine epilepsy is strongly exemplified in this case. It seems as if the determination to the head was removed; but, the necessary discharge by the catamenia not succeeding, a new morbid determination took place, occasioning the attack of phlegmasia; an occurrence by no means unusual."

II. In cases of epilepsy from dysmenorrhœa, the practice to be pursued must be somewhat different from that recommended in the foregoing observation.

We shall quote a short case, indicative of Dr. Prichard's views on this subject.

"Mary Hodge, admitted out-patient, May 11, 1818. A girl of fat and plethoric habit, twenty-two years old, who has been five years subject to epileptic fits. They always accompany the returns of the catamenia, and occur at the conclusion. She says, she has never missed having the fits at the usual period: never has more than one at a time. They attack her without any premonitory symptom. Bowels regular; pulse natural.—Venæ sec. fluant sang. ℥xvj. Pil. cath. o. n. Mist. cath. ter in die.

"*June* 3.—Complains of tenderness of the epigastrium: pain on pressure.—Pil. cath. o. n. Mist. menth. c. rheo. ter d.

"30.—Feels herself well.—Continue.

"*July* 3.—Repeat the medicines.

"10.—She has had no fits. The catamenia have been expected now two days. Formerly they were always regular to a day.

"17.—The catamenia have taken place without any return of the fits.—Continue.

"25.—She has had no fits: is quite well.—Continue the aperient medicines.

"I heard no more of her.

"*Observations.*—It is very remarkable that the evacuant measures adopted in this case were so soon effectual in interrupting, *at least*, the return of the epileptic fits, which had previously accompanied, in so unvarying a manner, the periods of the catamenia."

Of maniacal affections, connected with states of the uterine functions,—of their nature and treatment,—of puerperal mania,—of maniacal affections occurring at the period of menespausia,—all which points Dr. Prichard has touched upon in as many sections, we have no room left to say any thing. The author's views are clear, the inferences correct, and the cases in support of them interesting.

In chapter sixth, Dr. Prichard pursues his inquiry into epileptic and maniacal affections arising from metastasis, or the translation of morbid action from other structures to the brain. Among the most striking examples of this kind, the author enumerates those cases of metastasis in the head which follow the healing of old ulcers, or the disappearance of eruptive disorders. Next he mentions a similar effect produced by gout, rheumatism, and the inflammation of serous membranes. Dropsical inflammation, translated to the brain, is also considered as a source of convulsive or maniacal affections; and two cases are, finally, recorded by the author; in which disorders of the brain have occurred consequent on the removal of tumors. It is needless to observe, that a similar excitement to disease of the brain is occasioned by the suppression or cessation of habitual hemorrhages; and that the converse of what has, here, been noticed will not unfrequently occur, where maniacal affections are cured by the substitution of another diseased action, or, as it were, metastatically. The author has well illustrated the pathology of both these classes.

With regard to their treatment, Dr. Prichard is aware that, although the principal indications are very analogous, they are not always to be attained by similar means. His observations on bleeding, purging, and the use of mercury, will be read with interest.

By far the most important chapter in the volume before us, is, without doubt, the seventh, which treats of epilepsy and maniacal cases depending on a disordered state of the intestinal canal. Nor are the succeeding observations of the author, on what he has called the hepatic disease, worthy of less consideration. We wish our limits permitted us to enter into an analytical review of what Dr. Prichard has advanced in both these subdivisions of his work; for we feel confident that we should thereby render a great service to those practitioners who are not likely so soon to see the original. Unfortunately, we have already extended our analysis so far, that we could not, without encroaching on the other departments of our Journal, dilate farther on the same subject.

We have said enough, we think, to excite a desire in the profession to peruse the volume itself; and thus far we have served the cause of medical science. Dr. Prichard's observations seem to be derived from very extensive practice, and the simplicity with which the numerous cases illustrative of enteric epilepsy and enteric mania are narrated, must go far in commanding the confidence of his readers. It is proper to observe, that the details of all the cases brought forward by the author are extracted or abridged, principally, from memoranda and registers kept by the author himself, at St. Peter's Hospital and the Bristol Infirmary, and, in part, from a book of cases kept by the apothecary of the former hospital, Mr. A. Kift, now a medical officer in the navy.

The remaining subjects treated in this volume are, traumatic mania,—cerebral disease produced by physical agents,—local convulsions, or partial epilepsy,—convulsive tremor,—and somnambulism, or ecstasis.

The author informs us, that he looks forward to the publication of another volume, at some future time, on several disorders of the nervous system which are omitted in the present treatise, particularly on chorea, hysteria, and comatose affections. On some of these subjects he has collected a considerable number of illustrative cases; and we shall hail with pleasure the completion of a work, which, together with the more learned production of Dr. Cooke, goes far in placing the English bibliography of nervous diseases on a highly respectable footing.

---

**Art. II.**—*A Treatise on the Diseases of the Chest, in which they are described according to their Anatomical Characters, and their Diagnosis established on a new Principle by means of Acoustick Instruments. With Plates.* Translated from the French of R. T. H. Laennec, m.d. with a Preface and Notes, by John Forbes, m.d. Physician to the Penzance Dispensary, Secretary of the Royal Geological Society of Cornwall, &c. &c. 8vo. pp. xl. 437. T. and G. Underwood. London, 1821.

In the forty-third volume of our Journal, we entered at large into the consideration of Dr. Laennec's work; and we endeavoured, on those occasions, to give as copious and accurate an account of its contents as the nature of our publication would admit, and our sense of the importance of that work seemed to suggest. In announcing, therefore, a translation of that performance, our readers will not expect us to dilate, once more, on the great merits of the original, or to repeat what the most respectable of our contemporaries, as well as ourselves, have already said respecting its utility and superior claims to the attention of the profession. It will be sufficient for us to point out under what circumstances the present translation comes into the world; what degree of confidence the public may place in the translator; and in what manner he has discharged the important duty he has undertaken to perform.

Of Dr. John Forbes, personally, we know nothing: hence we shall not be suspected of bias either way, in judging of his abilities on the present occasion.

The principal motive, with Dr. Forbes, for undertaking the laborious task of translating Laennec's work, was the hope of rendering its most valuable contents accessible to the English reader. To effect this object with a greater chance of success, the method of translating the work adopted by Dr. Forbes has been to condense two volumes into one; by following a somewhat different arrangement from the original; and by, considerably, abridging the cases, as well as some part of the diagnostic details, though in a lesser degree.

With regard to the first of these deviations, we are decidedly of opinion that the majority of the English readers of Dr. Forbes's translation will feel the advantage of it. The arrangement he has adopted is grounded on pathological principles, which are, unquestionably, less precarious and less fleeting than those derived from diagnostic characters, dependent on a new mode of instrumental exploration. Thus, instead of having, as in the original, the diseases marshalled under four heads, according as they are recognized by the exploration of the voice, the respiration, the rattle, and the circulation, and the diagnosis of each disease conjoined and mingled with its description,—we find, in the first place, an entire separation of the descriptive from the diagnostic part; and, secondly, a subdivision of each of these, according to the respective localities of the diseases, as affecting the lungs, pleura, and heart.

"In its present condition, therefore, the work will be found equally available to the practitioner, as a system of pathological anatomy, whether he adopts or rejects the author's diagnostics; while, in the original form, these two departments were so interwoven with each other, that, without a previous study of the one, the other could not be well understood. In this respect, then, I consider both the author and the English reader as obliged to me; inasmuch as, without in the least altering either the facts of the pathology or the diagnosis, I have rendered them both much more accessible, and placed them in a clearer, closer, and more connected, point of view."

We are not sure, however, that the second deviation of Dr. Forbes from the original, namely, his having considerably abridged the cases, will meet with the same degree of approbation from his readers. There is only one excuse for the adoption of such a measure on the part of a translator, namely, the desire of avoiding "bulk and high price;" two very serious inconveniences, undoubtedly, to a large number of the profession. But a consideration of this nature weighs little against the disadvantage of having cases and histories of diseases, and, not unfrequently, diagnostic descriptions of them, curtailed of many, and, for aught we know, some of the most essential, of their characteristic features. Laennec's being strictly a book of reference,—a work which, like Morgagni's, will invariably be looked into, for the future, in cases of difficulty,—it would have been better to have introduced it to the English readers with its redundancies, rather than exhibit it shorn of its fair proportions. Who is there that, in cases of appeal to the high authority of the illustrious Italian pathologist, refers to an abbreviated edition of its imperishable work? Indeed, we find that Dr. Forbes himself began to feel the force of some such observations as these, when he got to the end of his labour; for, in an Appendix to his translation, he has added some cases, which he had left wholly unnoticed in the body of the work, and has given fuller details of some that he had too much abridged.

To judge of Dr. Forbes's sentiments from a perusal of his Preface, we must confess that they are such as to inspire confidence in him, not only as a faithful translator, but as an impartial and, to all appearances, a well-qualified judge of the validity of the new doctrines and practice which he has undertaken to communicate to his English brethren, from the French author. Dr. Forbes expresses his conviction of the great importance of morbid anatomy as a means of improving the practice of medicine; and he has, also, a moderate share of enthusiasm for the two modes of diagnosis proposed, by our continental brethren, in ambiguous diseases of the thoracic viscera,—*percussion*, and *mediate auscultation;* which latter mode, as our readers have before been informed, is peculiar to the author, of whose volumes Dr. Forbes has offered here a translation.

Mediate auscultation, as well as the instrument employed to carry it into execution, many, in this country, have affected to despise and laugh at. As it generally happens in such cases, those individuals who laughed and despised, did so before they had tried any experiments, or had seen the results of those that had been made by others. Not so Dr. Forbes: he acknowledges his limited practice in the use of the *stethoscope*, it is true; but he seems satisfied that it is by no means one of those idle inventions, which must soon fall into neglect, rather than call for commendation. On this point, however, we prefer leaving him to speak for himself.

"In respect to the general merits of mediate auscultation as a means of diagnosis, I am disposed to coincide in the high opinion of the author; due allowance being made for the natural partiality of a discoverer. My own experience, of course, has been, comparatively with that of the author, quite insignificant; and has, in every case but one, wanted the only sure seal of merit, morbid dissection: although, therefore, it would have been both unphilosophical and indecorous to have allowed the few results obtained by me, had they been opposed to those of M. Laennec, to weigh in any respect against the immense experience of that gentleman,—it must be satisfactory, at least, to know that they have, as far as they go, coincided with his in every respect. I am sorry to say, that it is only within the last few months that I have given the new methods of diagnosis a fair and continuous trial; and I am the more sorry, as I am daily more and more convinced of their extreme value. It is in chronic cases, more especially, that the method of M. Laennec will be found of the greatest advantage; and, exclusive of the unimpeached testimony of the author, I have no doubt whatever, from my own experience of its value, that it will be acknowledged to be one of the greatest discoveries in medicine, by all those who are of a temper, and in circumstances, that will enable them to give it a fair trial. That it will ever come into general use, notwithstanding its value, I am extremely doubtful; because its beneficial application requires much time, and gives a good deal of trouble both to the patient and the practitioner; and because its whole hue and character is foreign, and opposed to all our habits and associations. It must be confessed, that there is something even ludicrous in the picture of a grave physician formally listening through a long tube applied to the patient's thorax, as if the disease within were a living being that could communicate its condition to the sense without. Besides, there is in this method a sort of bold claim and pretension to certainty and precision of diagnosis, which cannot, at first sight, but be somewhat startling to a mind deeply versed in the knowledge and uncertainties of our art, and to the calm and cautious habits of philosophising to which the English physician is accustomed. On all these accounts, and others that might be mentioned, I conclude that the new method will only in a few cases be speedily adopted, and never generally. In all hospitals, however, both civil and military, and in the public services of the army and navy,—in all of which situations the above-mentioned obstacles to its employment scarcely exist,—I should hope that its adoption will be less tardy and partial. It is to them, especially, that it is adapted; it is in them that its merits can be put to the test; and it is to be hoped that, if its value is once acknowledged in them, no minor objections of mere inconvenience or formality will be permitted to effect its exclusion from general practice."

Dr. Duncan, junior, is one of those who have given the practice a fair trial; and he is said to have gained many proselytes among the students. His report of its merits is most satisfactory; and, as his experience has been considerable, and in a field favourable to correct observation,—affording frequent opportunities of proving, by morbid dissections, the correctness of previous opinions,—it is to he hoped that the invention will shortly receive, from his authority, a fresh title to the consideration of the medical public.

Dr. Duncan has informed Dr. Forbes that he has made great use of the stethoscope,* and that he is satisfied of its utility in facilitating the diagnosis of diseases of the chest. At first, the novelty of the measure excited much merriment, and more scepticism; but the accuracy of the diagnosis obtained by it soon compensated for, and removed, those hostile feelings.

As we are anxious to see Dr. Laennec's practice more generally adopted amongst us, we shall not apologise for making another ample extract from Dr. Forbes's prefatory remarks to his translation, in which he gives an account of the difficulties he at first encountered in the use of the stethoscope, and how he at last surmounted them; and where he, also, details a few necessary precautions and instructions for the right application of that instrument.

"In corroboration of Dr. Duncan's statement, I may also observe, that my first trials of the instrument were very unsatisfactory, from the doubtfulness and uncertainty of the results obtained. This, however, arose entirely from inexperience, and from not attending pro-

* For a description of, and mode of using, this instrument, see page 164 of the 43d volume of the *London Med. and Phys. Journal.*

perly to the directions given for using the instrument. All the uncertainty and apparent difficulty was soon removed by a little practice; and I speedily became convinced that the results obtained were of that distinct and precise character, to justify every expectation of advantage from their being attended to in the diagnosis of disease.

" In exploring the respiration, more particularly, we must be most careful to keep the funnel-shaped extremity of the instrument very exactly applied to the chest, by its whole circular edge, which can only be done by attending to the author's direction of holding it as we do a pen, and keeping the hand quite close to the patient's chest. We must never attempt to alter the perpendicularity of the instrument, so as to bring its extremity to suit the convenience of the ear, but must bring the ear to it; and we must be careful not to press, with any considerable degree of force, upon the instrument, yet, at the same time, apply the ear quite close to it, and with the meatus directly opposed to its canal. All these precautions are necessary on account of the moderate degree of the respiratory murmur, which, although in general extremely distinct, is often very low, and never, except in certain cases of disease, such as to be called loud, unless comparatively with its own habitual condition. From the same circumstance, the injunction of the author respecting the absence of all other noises in the chamber of the patient, is never to be forgotten; as talking, even in a whisper, in the room, or any noise in the street, from wheels, &c. will often effectually mask all perception of the sound we are attending to.

" In studying the action of the heart, also, I would suggest one caution respecting the statements of the author as to the extreme distinctness of the sounds. These, doubtless, are very distinct; but the quickness with which they succeed each other requires considerable care, and also experience, to obtain the necessary precision in recognizing them. This caution is, perhaps, the more necessary, as we are apt to forget the natural briefness of the heart's pulsations, while perusing the graduated and formal description of them given by the author. In respect of pectoriloquism, I would request attention to the varieties of it called *uncertain,* lest the non-perception of it in its very decided character, in cases where the conditions said to be productive of it may be presumed to exist, should produce scepticism respecting its occurrence at all."

With the following paragraph, from the succeeding page of Dr. Forbes's preface, we shall terminate our observations on the degree of confidence which the reader may place in him on the present occasion.

" In all cases, I must caution the young explorer not to be overhasty in condemning the practice. Let him recollect that the discoverer of the method has declared that it is only in hospital practice, or in a practice affording similar facilities of reiterated examination both before and after death, that it can be properly studied. Let him call to mind how many things, in his various studies, which seemed impracticable and false at first, further experience taught him to believe both easy and useful. Above all, let him never forget that the object proposed to be obtained by the new method, is the improvement of the diagnosis of a numerous class of most formidable diseases; an object, which, as it involves every thing that can be most valuable to a medical man, has a right to claim the most zealous and patient attention of every one who has at heart either his own professional success, or the welfare of his patients."

With regard to the manner in which Dr. Forbes has discharged the duty he has undertaken to perform, we have nothing but what is favourable to him, as a physician and a scholar, to offer. We are not strangers to the French language, and we can assure our readers that few works, written in that idiom, have had so much justice done to them by English translators as the one on which we have been commenting. To the translation, Dr. Forbes has added a few notes, in which he has noticed some of the principal English writers who have treated on the diseases described in Dr. Laennec's work.

The reader who, being at a distance from the metropolis, often judges of the importance of a work from a mere perusal of the index of its contents,—the copious and important nature of which, not unfrequently, induce him to purchase the book,—will not be sorry to see in what manner Dr. Forbes has arranged the abundant materials of his original, which he has now submitted, for the first time, to the consideration of the medical profession in this country.

The volume contains two parts: the one is entitled PATHOLOGY; DIAGNOSIS, the other. The former is subdivided into three books, treating of the diseases of the lungs, the pleura, and the heart and its appendages. The latter has no subdivision, and embraces the diagnostic consideration, or exploration, of many of the above complaints.

Under the head of Diseases of the Lungs, we have—

Phthisis Pulmonalis:—anatomical or essential characters; occasional changes; curability of phthisis; expectoration in phthisis; and vomicæ.

Peripneumony.

Gangrene of the Lungs.

Hæmoptysis, or Pulmonary Apoplexy.

Pulmonary Catarrh, or Bronchitis:—acute and chronic.

Dilatation of the Bronchia.

Emphysema of the Lungs.

Œdema of the Lungs.

Accidental Productions in the Lungs:—cysts; hydatids; osseous concretions, &c.; melanosis; and medullary tumor.

The book on Affections of the Pleura offers considerations on Pleurisy:—acute pleurisy; chronic pleurisy; contraction of the chest; gangrene of the pleura; and circumscribed pleurisy.

Hydrothorax.
Hœma-thorax.
Accidental Productions in the Pleura.
Pneumo-thorax.

—While that on Disorders of the Heart and its Appendages extends to the description of—

Diseases of the Heart:—hypertrophia; dilatation of the ventricles; dilatation, with hypertrophia, of the ventricles; dilatation of the auricles; partial dilatation of the heart; induration of the heart; softening of the heart; atrophy of the heart; fatty degeneration of the heart; ossification of the heart; carditis; ossification of the valves; accidental productions; polypus; excrescences on the valves; red colour of internal membrane; malformation of the heart; displacement of the heart.

Diseases of the Pericardium:—pericarditis; hydro-pericardium; and accidental productions.

Aneurism of the Aorta.

The second part opens with an account of the nature and discovery of Mediate Auscultation, and, after having presented a detail of the modes of exploring the voice, as well as the respiration and circulation, exhibits the peculiar stethoscopic signs by which we can recognize many of the diseases of the viscera already mentioned, or any simple morbid deviation in their structural functions.

After what we have had occasion to say of Dr. Forbes's performance, and the length to which we have deemed it proper to extend our observations on a simple translation, does not every reader anticipate our conclusion, that Dr. Forbes has, by the able manner in which he has performed his task, deserved well of the medical profession in this country?

---

Art. III.—*A Practical Essay on Ringworm of the Scalp, Scalled Head, and the other Species of Porrigo.* By Samuel Plumbe, Member of the Royal College of Surgeons of London, of the Medico-Chirurgical Society, &c. 8vo. pp. 104. London: T. and G. Underwood. 1821.

We always enter on our critical labours with much alacrity and pleasure, when we have to review a practical monograph, and prepare ourselves to disregard the style of composition for the more solid advantages of its matter; for we are rarely disappointed in our expectations of deriving such information from it as we can, with satisfaction, communicate to our readers, and such instruction as will enable us to confer benefit on our patients, from the practical knowledge we obtain in its perusal. Besides, when the mental energies of a man of talent are concentrated on the investigation of a particular subject, they seldom fail to throw an irradiation around it that will enlighten his fellow-men; so also, when the professional man of capacity directs his mind to the study of a particular disease, which we know he has ample opportunities of witnessing, and of exercising his judgment, reflection, and observations upon, we are naturally prepossessed with a persuasion that his pathological deductions will be drawn from conclusive facts, with close reasoning; and that the treatment recommended will be founded on experience, and the results of successful practice in numerous cases. Such appears to be the case with the unostentatious little essay before us, the author of which has endeavoured to convey to the public the knowledge of what he considers a speedy and uniformly-successful treatment of one of those obstinate cutaneous diseases, which, in many instances, has baffled the skill and judgment of our predecessors.

The author excludes porrigo larvalis, or crusta lactea, from his consideration, because "it is not in any case contagious," and differs in other respects from the species porrigo; and states that, "so far as experimental inquiry has gone, it appears probable that porrigo scutulata, furfurosa, and lupinosa, only, are the results of one specific contagion: and the points in which these differ from each other are of very small importance. The porrigo *decalvans* is of such rare occurrence as to make it difficult to form a decided opinion of the manner of its production." The experience of the author does not enable him to enter on the practical consideration of the latter species. Of the porrigo *favosa*, he observes, "that in no case does it appear, by infection, to produce either of the other species." "Whether the matter secreted be inserted under the cuticle, or simply applied by friction, the same *favous* pustule is produced as the original, which goes through its course, and is not found to show any of the obstinacy of the scutulata, the dry inactivity of the lupinosa, or the morbid secretion of cuticle which distinguishes the furfuraceous disease."—P. 27-29.

In chapter I. the author offers some preliminary remarks on the origin of the hair, and its influence in diseases situated on the scalp. On the former subject he infers, "there can be no doubt that the hairs have their origin completely beneath the under surface of the cutis of this part, and derive their support from the adipose membrane here disposed, and apparently having a particular arrangement for this purpose." On the latter subject he observes, "I am particularly desirous of impressing upon the attention of my readers the fact, that the scalp is pierced by the hair, and has little or no share in its production or nourishment. Reasoning from analogy, we should be justified, by this consideration only, in concluding that the latter *may* possess, when the former is in a state of disease, all the properties of extraneous substances. As regards the common ring-worm of this part, it will be uniformly found evincing these characteristics, in the mildest, as well as most severe, forms of the disease."

Chapter II. is on Porrigo Scutulata, or ring-worm of the scalp; of which he gives an accurate description.

"The symptom by which this disease is usually first discovered, is the falling-off of the hair of the part. The attention being attracted by this occurrence, the scalp appears, on examination, to have assumed a somewhat scurfy and slightly reddened appearance. The hair remaining on the diseased part is thin, and irregularly scattered over it; the greater portion appearing to have been removed by the roots, while some have been broken off near to the scalp, the roots of which still retain their situation. Those which remain apparently growing on the part will be found to drop off on friction, or to have, on being pulled, scarce any hold on the scalp. In the majority of cases, at their first commencement, these will be the only appearances noticed. The existence of the minute straw-coloured pustules, denominated *achores*, does not appear necessary to constitute the disease, as they are not seen for a short time later, and not till after some degree of itching and irritation of the part has been felt.

"The achores, usually, soon make their appearance after the hair begins to fall off. The itching and irritation commencing at the same time, the child, who is the subject of it, soon ruptures a few of them, and, spreading by the frequently-repeated application of the nails to the spot, their contents cover the adjacent parts of the scalp,—extends the disease, with great rapidity, upon it,—the same destruction of the hair, and subsequent pustulation, marking its progress.

"When pustules are noticed, they are uniformly found with hairs growing through them; and, if the disease has existed for a considerable length of time, and destroyed the greater part of the hair of the part, such pustules are found proportionately reduced in number; but, still surrounding the few straggling hairs which remain, each single minute pustule appearing to be dependent on the hair in its centre. If the hair be completely eradicated from the spot where the disease first appears, the skin assumes an apparently healthy character: the disease, as regards this particular spot, may be said to have exhausted itself. When the hair has grown again, a recurrence of the pustules may take place, and again destroy it. The specific irritation, constituting the essence of the disease, is diffused in a circular areola on the spot to which the infectious matter is first applied; and in the rapid extension of the mischief after pustulation has commenced, the same figure is preserved, or pretty nearly so."

The above description corresponds, in substance, with those of Drs. Willan and Bateman, except that the latter do not notice the falling-off of the hair, and the scurfy and reddened appearance of the scalp previously to the formation of pustules. If the disease be neglected, "the secretion from the pustules is allowed to accumulate, and scabs are formed of it upon the surface, which, being confined by their adhesion to the remaining hairs, become a means of further accumulation of this irritating fluid. The inflammatory action of the part [is] materially increased in this way: the quantity of fluid secreted, and the means of extension of the disease, receive, likewise, a proportionate increase; and it is this state of the disease which appears to be understood by the term Scalled Head. In a still more aggravated form of the disease, the accumulation of scabs, the offensiveness of the odour of the part, and the myriads of pediculi creeping over it, render it so disgusting as to discourage, very commonly, any earnest attempts at relief: and the patient (in the very lower [lowest] ranks in life,) is abandoned to the ravages of the disease."

There is a curious fact, mentioned at p. 49, relative to its propagation, where the author observes, that, "when the diseased surface is in a very irritable state, the application of the matter to a sound part of the same scalp has often failed to produce the disease; while the same experiment on a healthy scalp, with the same matter, has been followed by this effect."

The pathology of porrigo scutulata is summed up in these words:—"The disease may be defined to be inflammation of a specific character, affecting the solid structure of the cutis. It will be recollected, that pustules only occur where hairs exist, and that each pustule has, usually, one in its centre; and, bearing in mind what has been already stated as to the probable influence of the hair on the scalp, where the cutis is actively inflamed, we may be warranted in the conclusion, that *the specific irritation of the disease obtains an accession of strength from the local influence of the hair, where it penetrates the scalp; and that the formation of a pustule is the consequence of such accession.*"

In chapter III. the author describes his mode of treatment, which is exceedingly simple, and, by his account, very efficacious. "The first important step," says he, "is *the removal of any hair remaining on the part, which may appear to come away easily, and without pain to the patient.*" This is done by a pair of forceps. The second is, to effect the discharge of the contents of as many of the pustules as possible, by pinching up the skin between the finger and the thumb. "This having been effected, and the part washed with warm water and soap, some astringent application, possessing *the power of taking from the secretion its infectious properties, and, at the same time, sufficiently powerful to constringe the vessels from which it flows, and lessen its quantity, may be made use of.*" A solution of sulphate of copper was employed, in some cases, for this purpose. The author believes, however, the object to be more completely accomplished by rubbing this preparation, in a finely-powdered state, on the part, and then washing it off. This should be done every morning; and, after two or three repetitions of this application, no fresh appearance of pustules takes place, and the circle of the disease is marked by small thin scabs, of a darkish colour. These scabs separate in a few days, and leave a shining, red, and irregular surface, which gradually loses its inflammatory character, having now and then a little scurf forming on it, till the new hair begins to appear. The hair afterwards becomes strong and healthy, in from six to twelve weeks. In the more aggravated forms of disease, the author avails himself of fomentations, poultices, and pressure by means of the adhesive straps and bandages, with the cold lotion in conjunction.

The necessity of internal remedies, in the milder cases, is doubted by our author; but, where severe constitutional irritation may be expected in the severe cases, and a feverish state of the system is present, constitutional treatment should, he thinks, be directed to subdue those symptoms; in which opinion we perfectly coincide with him.

Chapter IV. treats of Porrigo Favosa, which consists of an eruption of the large, soft, straw-coloured pustules, denominated "favi." The description is taken from Dr. Willan. This disease generally occurs in robust and apparently healthy constitutions; and a too-liberal allowance of animal food is said to produce it frequently, where infection is not the source. In the management of this disease, the author observes, there will be more frequently found a necessity for depletion and alteratives, than for the tonics which have been recommended. Applications which allay irritation are useful auxiliaries, and these, together, will be found adequate to the necessities of any case of the disease not occurring on the scalp. In protracted cases, such as are described by Alibert and others, where the disease is aggravated by myriads of vermin, and the accumulation of weeks and months of the secretion of the part, it is absolutely necessary to remove such collections of filth, as well as the hair, by a continued soaking and ablution with warm water and soap, and by shaving the parts with a razor. Fomentations and poultices are then necessary to subdue the inflammatory action of the vessels of the part; and, when this has been effected, a little attention to the general health is sometimes all that is necessary to the cure. After irritation has been allayed, it sometimes happens that the effusion of a viscous fluid is still kept up: in such cases, an effectual application will be found in a solution of argent. nitr. ℈j. in aq. distill. ℥j., or cupri sulph. ʒj. in aq. ferv. ℥ss., which should be applied, with a camel's-hair pencil, to the abraded surface, two or three times a-day, until the discharge ceases. The extraction of the hair by the forceps is not recommended in this species.

Chapter V. treats of Porrigo Furfurans; and, as the author is of opinion that the general resemblance of porrigo scutulata and furfurans is almost sufficient to identify them, and as no unsuccessful practice appears to result from the adoption of the principles of treatment, in the management of the latter, which has been laid down for the former, we shall not enter into any detail on this species. We may, however, observe, that there is a scurfy state of the scalp subsequent to cases of long duration, which is corrected and removed by a weak solution of lunar caustic, and a lotion of diluted alcohol, alternately employed.

In chapter VI. on Porrigo Lupinosa, the author states it to be his opinion, that this species of porrigo is only a modification of porrigo scutulata; and observes, that its cure is easily effected by adherence to the principles of treatment pointed out in his consideration of porrigo scutulata.

This essay is very creditable to the talents and humanity of its author, and may be highly beneficial to his fellow-creatures, and useful to his professional brethren. We are sure, however, he will excuse us for observing, that the printer and compositor have been so negligent and incorrect in the punctuation, as to frequently embarrass the reader by obscuring the text.

### DIVISION II.

### FOREIGN.

**Art. IV.—*Lezioni di Terapia speziale sulle Infiammazioni, e Rendiconto Clinico, di* V. Mantovani, &c.—*or, Practical Lectures on Inflammatory Diseases; and Clinical Report.* By V. Mantovani, Professor in the University of Pavia. 3 vols. 12mo. pp. 428, 448, 400.—1820.**

We have heard much of the inefficacy of the practice of foreign physicians. Dr. Clarke, of Rome, who, among many excellent things, we venture to say, has advanced not a few which would have been better out than in his book of Notes on Climate and Hospitals, is one among those who are pretty forward in censuring the milk-and-water—the *tisanne*, practice of continental physicians. Observations, of a character similar to those of Dr. Clarke, are for ever made and repeated; so that, were we to believe some of the writers on this side of the Channel, the wretched populations of France, Germany, and Italy, must be supposed to be perpetually feeling the dire effects of being treated by ignorant and inefficient practitioners; and to exhibit Bills of Mortality, such as to appal the more fortunate Englishmen. To prove that the case is not so, we need only refer to facts, well known to the majority of our readers. The laws of population follow precisely the same progress on the continent that they do amongst us; and those who fall victims to disease in France or Italy, bear exactly the same proportion that we are taught to believe the dead bear to the living in England. The fact is, that Dr. Clarke did, with regard to foreign nations, what Professor Tommasini, we understand, has done with regard to this country; and both have done wrong. Ere they could have had time to appreciate, duly, the character, value, and correctness, of the medical practice on which they had individually undertaken to report, they published hasty conclusions of their own as matters of fact. Tommasini, who has taken upon himself, since his return to Italy, to inform his countrymen of the present state of physic in England, has committed a number of *bevues*, (an excellent word for the occasion,) for which Dr. Clarke had already set him an example, with regard to the practice of physicians in Italy.

The three volumes before us come from a distinguished disciple of the Rasorian, or Italian school, and may be considered as giving a fair representation of the modern doctrine and practice in the north of Italy. We have compared them with the most esteemed works of other authors in France and England on the same subjects, and we do not find that the Italian professor has reason to blush at the comparison.

Professor Mantovani is not a man of mere words. We have no prettily-arranged classification of the diseases on which he treats, in his volumes; no attempts to assimilate complaints that have nothing in common with each other; no desire to simplify that which nature has presented to us in a complicated form. The author simply relates things as he has himself seen them,—describes those inflammatory diseases which he has had occasion to treat,—and gives an account of the result of the treatment he has employed to combat them successfully.

The FIRST LECTURE contains an historical survey of the various opinions advanced by writers of all ages and nations on the nature of inflammation in general. All these opinions being, necessarily, conjectural, the practitioner can derive no benefit from an acquaintance with them. He then describes the *phlegmon*, its causes, symptoms, and termination. The diagnosis, prognosis, and treatment, of this complaint are also fully detailed.

We next have an excellent monograph of *erysipelas* in the SECOND LECTURE, with particular reference to that of the face. The author is not disposed to admit the commonly-received pathogeny of this disease. In the treatment of it, he considers bleeding as the principal remedy.

LECTURE THIRD treats of *otitis*, *glossitis*, and *coriza*. The second of these inflammatory complaints has been well described by Sennertus, and Tissot, who experienced it himself, though in a slight degree; but none have dilated more upon it, or made the disease better known to the profession, than Raggi, who had repeatedly witnessed its formidable effects, during its epidemic prevalence, ten years ago, at Modena. Inflammation of the tongue may be the result of some injury done to that organ; but Signor Mantovani, in this place, intends to speak of the idiopathic species, of which he has given an admirable description.

The subject of *angina*, and its varieties, occupies the FOURTH LECTURE; in which some excellent observations on croup are also inserted, with an account of the doctrines and curative methods of Drs. Venturi, Double, and Albers, in that disease. Professor Mantovani divides *bronchitis* into three species: the hyper-acute, the acute, and the chronic. These are described at full length in the FIFTH LECTURE; and Dr. Badham's opinions are frequently referred to.

By far the most extensive part of the first volume, however, is that which is taken up with a description of what the professor has denominated *pulmonitis*. The varieties of this affection, admitted by the author, will, we doubt not, be deemed fanciful rather than to exist in reality. Yet the greater part of the continental physicians seem to share in the professor's opinion. This lecture is highly important, and extends to upwards of 160 pages.

*Carditis* is treated of in the SEVENTH LECTURE; and the singular case, recorded by Frank, of Vienna, illustrative of the rapidity with which this disease will sometimes destroy life, is aptly repeated by the author.

The second volume offers nine lectures on subjects of the greatest importance to practitioners: these are—1. Encephalitis; 2. Mielitis; 3. Diaphragmitis; 4. Peritonitis; 5. Gastritis; 6. Enteritis; 7. Epatitis; 8. Pancreatis; and 9. Splenitis. In treating of gastritis, and of ulceration consequent on that disease, the author takes the opportunity of relating the case of the late Professor Brugnatelli, the chemist, in whose stomach a perforation was found, one inch in diameter, scirrhous at a short distance from its margin, where the appearances of active ulceration were very evident: yet the patient had never complained of any pain or uneasiness, during life, in the region of the stomach; nor had he, otherwise, exhibited symptoms of gastric inflammation.

In the third volume we find a continuation of the author's description of various other inflammatory complaints: these are Nephritis, Cistitis, Metritis, Psoitis, Rheumatism, and Arthritis, or Gout. The gastro-hepatic pathology of the latter disease, so much in vogue in this country, does not appear to be admitted by continental physicians; but in these matters they cannot, certainly, be considered as good judges of the question, since it is but seldom that gout offers itself to their consideration in its genuine form.

One half of the third volume is taken up with the Clinical Report of the author's practice, during one scholastic year. Of 183 picked cases of acute and chronic diseases, admitted, chiefly, for the instruction of the university pupils, and selected from among the worst out of a public hospital, 159 were discharged cured, and eighteen died: the remainder were either relieved or remained under treatment. The largest number of patients were those affected with pulmonitis: the mortality among these was twenty-four in a hundred. Next came the typhoid patients, three of whom, only, died out of thirty-three. We suspect that few hospitals can offer more satisfactory results.

---

